# Rituximab-Associated Inflammatory Progressive Multifocal Leukoencephalopathy

**DOI:** 10.1155/2016/8915047

**Published:** 2016-11-14

**Authors:** Chandra Punch, Christina Schofield, Penelope Harris

**Affiliations:** Madigan Army Medical Center, Tacoma, WA, USA

## Abstract

Progressive multifocal leukoencephalopathy (PML) is a rare disease of the immunosuppression that results from neurotropic invasion of the JC virus which leads to demyelination of oligodendrocytes. Immune reconstitution inflammatory syndrome (IRIS), on the other hand, is a condition of inflammation that develops as the immune system reconstitutes. This case report describes a case of a 35-year-old HIV-negative male who presented with three weeks of right lower extremity paresthesias as well as right upper extremity apraxia. He was diagnosed with PML complicated by IRIS secondary to Rituximab, which he had completed four months prior to presentation. Despite the condition's poor prognosis, the patient recovered with only minor deficits.

## 1. Introduction

Progressive multifocal leukoencephalopathy (PML) is a rare disease resulting from the neurotropic invasion of the JC virus in the setting of immunosuppression [1]. In patients with PML, immune reconstitution inflammatory syndrome (IRIS), a marked inflammatory condition in response to an underlying infectious process, can develop as the immune system reconstitutes. While the return of immune function is essential to recovery from PML, overactivation leading to IRIS can potentially be fatal [2]. We discuss the management of a patient previously treated with Rituximab with PML complicated by IRIS.

## 2. Case Report

**Figure 1:**
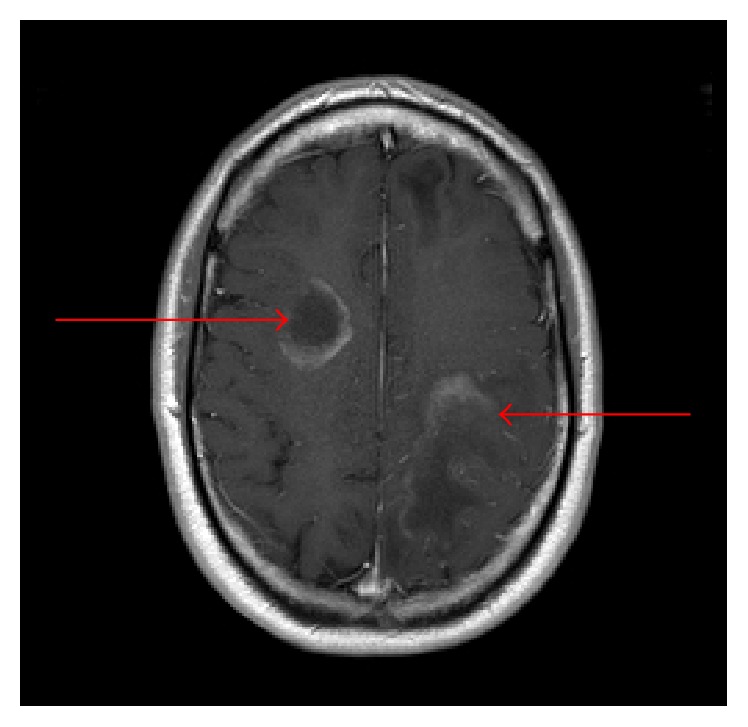
Brain MRI with bilateral peripherally enhancing white matter lesions representing signs of active demyelination.

**Figure 2:**
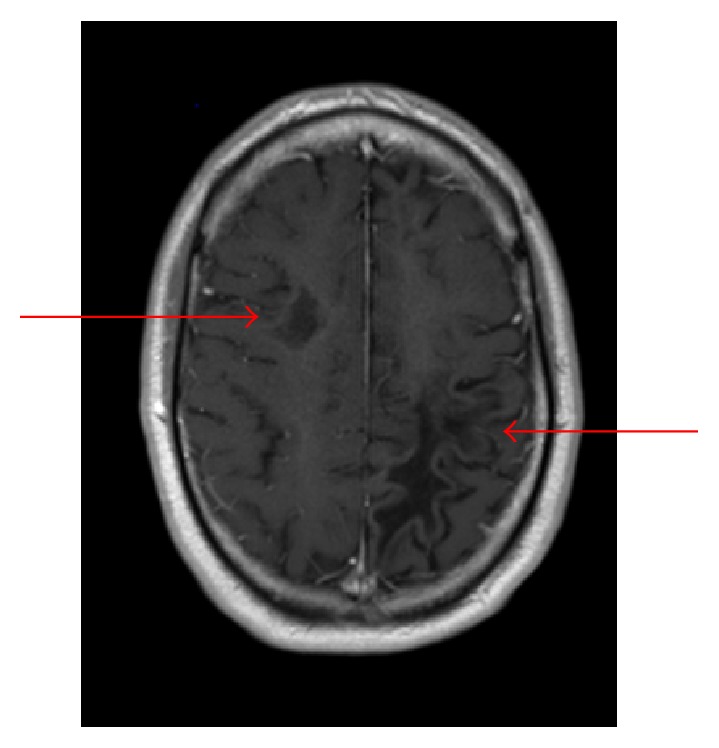
Interval improvement in enhancing white matter lesions consistent with clinical improvement.

A 35-year-old HIV- (human immunodeficiency virus-) negative male presented with three weeks of right lower extremity paresthesias and right upper extremity apraxia. His history was significant for Ann Arbor stage IV follicular lymphoma, in remission after initial Bendamustine and Rituximab, followed by maintenance Rituximab completed four months earlier. Physical exam revealed right upper extremity apraxia and weakness. Head CT revealed new bifrontal multifocal white matter hypoattenuation, while MRI brain (see Figure 1) revealed bilateral peripherally enhancing white matter lesions and signs of demyelination. Cerebrospinal Fluid (CSF) PCR was positive for the JC virus. Subsequent bone marrow biopsy and PET scan were negative for lymphoma. Flow cytometry revealed a CD4 count of 322/mcL. His presentation was consistent with inflammatory PML during immune reconstitution. Plasma exchange was deferred given the patient had not received Rituximab for over 4 months. Initial treatment with Mirtazapine 15 mg was started. However, the patient continued to develop enlarging lesions on imaging and worsening neurologic status including new onset aphasia. After expert consultation, he was then given Maraviroc, a small molecule CCR5 antagonist used to treat HIV, 300 mg twice daily without improvement, as well as a trial of methylprednisolone 1000 mg IV for three days without improvement. His clinical course was further complicated by bilateral retinal hemorrhages, and papilledema was thought secondary to IRIS. Due to the progression of disease, the Maraviroc was discontinued. He proceeded to intensive neurologic rehabilitation for patients with traumatic brain injury. He continued to dramatically improve neurologically; however he did not return to his premorbid baseline. Remarkably, he has since cleared the CSF of the JC virus on follow-up labs and shown substantial interval imaging improvement on recent MRI (Figure 2).

## 3. Discussion

The JC virus is mostly acquired during childhood or adolescence and remains in various organs including kidneys, bone marrow, and lymphoid tissue [2]. In the setting of immunosuppression, the JC virus can enter the brain and infect oligodendrocytes and astrocytes resulting in demyelination and PML. PML primarily presents with symptoms of motor deficits, visual disturbances, and cognitive impairment. MRI usually reveals hyperintense lesions on T2 weighted and FLAIR sequences located primarily in the subcortical white matter of the cerebral hemispheres and cerebellar peduncles [3]. At this time, there is no standard successful treatment for PML from JC virus. Drugs including Cytarabine, Cidofovir, Interferon, Mefloquine, and Mirtazapine have been studied without proven benefit [2].

While the development of immune reconstitution is essential for clearance of JC virus and recovery from PML, IRIS can occur as a complication of immune reconstitution leading to possible death. While guidelines for diagnosis exist in HIV-positive patients undergoing therapy, the diverse clinical presentation prevents strict diagnostic criteria for both HIV-positive and HIV-negative patients. Furthermore, there are no specific tests to establish an IRIS diagnosis. In IRIS, gadolinium enhancement on MRI, an atypical finding in PML, in association with worsening neurologic symptoms, can aid in diagnosis. While use of glucocorticoids is currently the common treatment, little benefit has been proven and steroids can potentially limit JC viral clearance. PML may be more prevalent in patients with low CD4 counts; however once again after expert consultation use of IL-7 was deferred since the patient's CD4 count was >200. The benefit of Maraviroc in preventing further complications of IRIS through the inhibition of CC5 dependent immune trafficking into the central nervous system has been noted in case reports. Our patient did not benefit from steroids or Maraviroc [4]; however he did have dramatic improvement with aggressive neurologic rehabilitation bringing him close to his premorbid baseline.

In conclusion, the development of IRIS that cleared the CSF of JC virus despite the patient's poor prognosis, related to hematologic malignancy and Rituximab-associated disease, makes his case unique [3]. His improvement highlights the potential for clinical improvement from IRIS-associated disability and the importance of optimizing patient immune function in conjunction with intensive neurologic rehabilitation in individuals with this disease [2].
